# Habitual fat intake predicts memory function in younger women

**DOI:** 10.3389/fnhum.2013.00838

**Published:** 2013-12-11

**Authors:** E. Leigh Gibson, Suzanne Barr, Yvonne M. Jeanes

**Affiliations:** ^1^Department of Psychology, Whitelands College, University of RoehamptonLondon, UK; ^2^Diabetes and Nutritional Sciences Division, King's College LondonLondon, UK; ^3^Department of Life Sciences, Whitelands College, University of RoehamptonLondon, UK

**Keywords:** cognition, memory, hippocampus, saturated fat, unsaturated fat, insulin, women

## Abstract

High intakes of fat have been linked to greater cognitive decline in old age, but such associations may already occur in younger adults. We tested memory and learning in 38 women (25 to 45 years old), recruited for a larger observational study in women with polycystic ovary syndrome. These women varied in health status, though not significantly between cases (*n* = 23) and controls (*n* = 15). Performance on tests sensitive to medial temporal lobe function (CANTABeclipse, Cambridge Cognition Ltd, Cambridge, UK), i.e., verbal memory, visuo-spatial learning, and delayed pattern matching (DMS), were compared with intakes of macronutrients from 7-day diet diaries and physiological indices of metabolic syndrome. Partial correlations were adjusted for age, activity, and verbal IQ (National Adult Reading Test). Greater intakes of saturated and trans fats, and higher saturated to unsaturated fat ratio (Sat:UFA), were associated with more errors on the visuo-spatial task and with poorer word recall and recognition. Unexpectedly, higher UFA intake predicted poorer performance on the word recall and recognition measures. Fasting insulin was positively correlated with poorer word recognition only, whereas higher blood total cholesterol was associated only with visuo-spatial learning errors. None of these variables predicted performance on a DMS test. The significant nutrient–cognition relationships were tested for mediation by total energy intake: saturated and trans fat intakes, and Sat:UFA, remained significant predictors specifically of visuo-spatial learning errors, whereas total fat and UFA intakes now predicted only poorer word recall. Examination of associations separately for monounsaturated (MUFA) and polyunsaturated fats suggested that only MUFA intake was predictive of poorer word recall. Saturated and trans fats, and fasting insulin, may already be associated with cognitive deficits in younger women. The findings need extending but may have important implications for public health.

## INTRODUCTION

Long-term maintenance of optimal cognitive function is becoming increasingly important, given the shift in demographics toward a growing elderly population. There is accumulating evidence for the importance of lifestyle factors in maintaining cognitive function into older age (Gillette Guyonnet et al., 2007; Lee et al., 2009). A recent longitudinal epidemiological study found evidence for cognitive decline in middle-aged adults: a cohort of 10,308 British civil servants aged 45–70 (known as the Whitehall II study) was assessed for cognitive function on three occasions over 10 years (Singh-Manoux et al., 2012). Cognitive decline was apparent in all age groups, even in those aged 45–49 at baseline; moreover, the effect was probably underestimated due to practice effects. Such cognitive decline in a relatively healthy group is most likely to be due to lifestyle factors including diet, perhaps interacting with genetic susceptibility.

Experimental studies in rodents have found that animals fed diets high in saturated fat for several weeks show impairments in learning and memory (Murray et al., 2009), and independently of obesity (Valladolid-Acebes et al., 2011). Various changes in the brain, particularly in the hippocampus, may underlie these effects, including a reduction in neurogenesis, increased inflammatory activity, altered blood–brain barrier permeability, reduced neuronal plasticity, and decreased hippocampal insulin sensitivity (Molteni et al., 2002; Kanoski et al., 2010; McNeilly et al., 2011; Davidson et al., 2012; Pancani et al., 2013). In human beings, diets high in saturated or trans fats, and low in polyunsaturated fats (PUFAs) have been linked with poorer cognitive function in older populations, both in cross-sectional epidemiological studies (Kalmijn et al., 2004) and in longitudinal studies in which cognition was tracked for several years (Morris et al., 2004; Gillette Guyonnet et al., 2007; Okereke et al., 2012). This includes the finding that the fat intake of 40- to 60-year-olds predicted cognitive function 20 years later, with relative deterioration associated with saturated fats and improvements associated with unsaturated fat intake (Eskelinen et al., 2008).

High circulating blood cholesterol has been linked to poorer cognition in some studies (Gendle et al., 2008), but findings are inconsistent and suggest that population cholesterol-lowering *per se* should not be a priority for cognitive function (Solomon et al., 2009; Reynolds et al., 2010).

Although saturated and trans fats have been linked to impaired cognition, conversely several observational studies suggest associations between higher intake of PUFAs, especially n-3 fatty acids (Kalmijn et al., 2004), but also n-6 fatty acids (Roberts et al., 2010) perhaps by displacing intake of saturated fat (Devore et al., 2009), and better cognition or protection from cognitive decline. These essential fatty acids are known to be critical for optimum neuronal membrane function, and their intake during pregnancy benefits cognitive development of the offspring (Helland et al., 2003; Daniels et al., 2004). More recent studies also support beneficial effects of higher monounsaturated fats (MUFA) on cognition: a 3-year follow-up study of 482 women aged at least 60 found that higher MUFA (assessed by food frequency questionnaire, FFQ) was associated with better memory and visual learning, but there was no association with saturated and trans fats, after adjusting for confounders including energy intake (Naqvi et al., 2011). In another prospective study of older Italians, baseline intakes of PUFA and MUFA (FFQ assessed) were associated with protection against cognitive decline over an 8.5-year follow-up, although total energy intake was similarly protective (Solfrizzi et al., 2006). In a randomized controlled trial in 522 elderly Spanish men and women with cardiovascular risk factors, one group received a low-fat control diet, while two other groups received Mediterranean-style diets, one of which was supplemented with extra-virgin olive oil, and other with mixed nuts, both of which are rich in MUFA (PREDIMED-NAVARRA trial; Martinez-Lapiscina et al., 2013). Cognitive function was assessed after 6.5 years of dietary intervention: in fully adjusted analyses, cognitive performance was found to be superior in both MUFA-rich dietary intervention groups compared to the control diet group.

However, not all studies support a beneficial effect of MUFA on cognitive decline: in 2002, results from a large 6-year prospective study of 5,395 Dutch adults (the Rotterdam Study), which included a semi-quantitative FFQ, and monitoring for dementia, did not find any association between either high intakes of saturated and trans fats, and cholesterol, or low intakes of MUFA and PUFA, and risk of developing dementia, after adjusting for likely confounds (Engelhart et al., 2002). Yet, only 197 participants (3.6%) had been diagnosed with dementia over that period, and more subtle declines in cognitive function were not assessed. In another large epidemiological study in the USA (Nurses’ Health Study; Samieri et al., 2013) in which women aged at least 70 were followed over 6 years, adherence to a Mediterranean diet, and MUFA:saturated fat ratio, were not significantly associated with cognitive decline. Furthermore, one recent report of 1528 60- to 64-year-old Australians assessed over 4 years found that the Mediterranean diet was not protective of cognitive decline, but instead, higher MUFA was actually associated with greater decline (Cherbuin and Anstey, 2012).

The evidence for chronic effects of diet on cognition has focused on the elderly, largely (though not always) relying on simple global cognitive tests (e.g., the Mini Mental State Examination – MMSE) that can be applied to substantial elderly populations and detect clinical cases or mild cognitive impairment. Moreover, nutrient intakes in these studies are usually assessed by FFQ, which are not accurate for measurement of absolute nutrient intakes (Flood et al., 2004). Given that detectable cognitive decline is thought to result from changes taking place over 20–30 years (Launer, 2005), it is possible that chronic dietary factors may already be affecting the cognition of younger adults. Thus, the question arises whether relationships between dietary fats, blood lipids, and cognition could be detected in younger ostensibly healthy populations, if more sensitive domain-specific cognitive tests and accurate dietary assessments, or trial designs, are used. Recently, two relevant experimental studies from the same group have been conducted in younger adult men: Edwards et al. (2011) asked 20 men aged 25–45 (mean age = 36 years) to eat a ketogenic very high-fat low-carbohydrate (Atkins-style) diet (74% energy as fat; 1.5% carbohydrate) for 7 days, following 3 days on a low-fat (17%) high-carbohydrate diet. Cognitive function over a range of domains was assessed by a sensitive computer-based test battery before and after this diet. There were no changes in memory function, but complex and simple reaction times were slightly longer after the high-fat diet, and calmness and alertness deteriorated. However, these results are difficult to interpret due to the absence of a parallel control group.

In a subsequent study with an improved randomized controlled cross-over design, 16 younger men, aged 19–28, ate a 70% fat, 4% carbohydrate diet for 5 days, and, on another 5 days, an equicaloric low-fat diet of 24% fat and 50% carbohydrate (both diets were high in protein, at 26% of energy), with a 2-week wash-out period in between, as well as 3 days on a standardized diet prior to each test diet (Holloway et al., 2011). Compared to the low-fat diet, the high-fat diet resulted in deterioration of focused attention and speed of memory retrieval, as well as slower information processing, although other measures of attention and episodic or working memory were not significantly affected. Mood was unaffected in this study. Similarly, in healthy overweight adults, after one week on a high-fat, low-carbohydrate weight-reducing diet, memory was impaired compared to participants following a more balanced reducing diet, though speed of responding on a vigilance task was faster (D’Anci et al., 2009). In contrast, a 6-week low-carbohydrate ketogenic diet improved memory relative to a high-carbohydrate diet, in elderly participants with mild cognitive impairment (Krikorian et al., 2012).

A limitation of those studies is the use of extreme differences in fat content, and the lack of information on the type of fat, although given the high protein content, it is likely that the high-fat diets were rich in saturated fats. It is also possible that this short term impairment of cognition would not occur over a longer dietary adaption period. One way to address this is to assess habitual diet and examine relationships between intake of fat type and cognition. This approach was taken in a study testing a heterogeneous sample of 498 men (32%) and women (68%) aged from 17 to 66 (mean = 20.5), with body mass index (BMI) ranging from 15 to 40 (Francis and Stevenson, 2011). Cognitive tests were chosen to be sensitive to hippocampal (or medial temporal lobe) function (story recall and verbal paired associate learning) as well as a digit span test included as a control for attention. Relevant aspects of habitual diet were assessed by a bespoke FFQ that included 24 typical dietary items known to contribute high levels of saturated fat or refined carbohydrate or both (Dietary Fat and Sugar Short Questionnaire, DFS-SQ; Francis and Stevenson, 2013). In regression analyses adjusting for potential confounders, there were weak but significant associations (0.8–2.9% variance explained) indicating that higher saturated fat and refined carbohydrate intake predicted poorer performance on the memory tasks. In a second study, two groups (*n* = 16 per group) were recruited based on high and low scores on the DFS-SQ, but otherwise matched for confounders such as age, sex, BMI, and verbal IQ. A larger range of cognitive tests was employed; nevertheless, significant differences were restricted to story recall and visual pattern reproduction. Verbal associate learning scores did not differ between groups, nor did a range of other cognitive tests assessing attention and executive function.

Limitations of these latter studies are that effects of fat and carbohydrates were combined, and differences in energy and other nutrient intakes were not accounted for. In particular, participants consuming greater quantities of the sort of energy dense foods assessed by the DFS-SQ may have higher energy intakes, albeit BMI and activity ranges were apparently matched. The possible importance of overall energy intake for memory function was demonstrated in an experimental study in healthy elderly men and women stratified by age, sex, and BMI into three 3-month dietary intervention groups: caloric restriction (CR; *n* = 20), enhanced intake of unsaturated fats (UFA; *n* = 20), and usual diet control (CON; *n* = 10; Witte et al., 2009). Cognitive function was assessed before and after the 3-month trial using a verbal recall test, a digit span test of attention and a working memory (trail making) task. Although self-reported dietary measures did not show a significant reduction in energy intake in the CR group, they showed significant weight loss, indicative of actual energy restriction. The UFA group showed a significant increase in unsaturated fats to saturated fat ratio, which is independent of energy intake. Effects were specific to the verbal recall test: CR showed significantly improved memory after the 3-month trial, whereas scores for the other two groups did not change. In sub-analyses of nine participants in the CR group who had weight loss >1 SD above the mean weight loss for the control group, fasting insulin, and the inflammatory marker C-reactive protein levels were strongly inversely correlated with memory performance. No benefits were found for the UFA group. Interestingly, similar results were found when obese women were put on a 50% energy reduction diet for 15 weeks: while there were no changes in tests of attention, verbal recall was improved (Kretsch et al., 1997). However, simple reaction time was slowed after energy restriction in this study.

Therefore, despite some intriguing evidence suggesting the importance of dietary fat type for cognitive health, there are inconsistencies, and even opposing effects, in the literature. Thus, the present study was designed to capture reliable dietary data (7-day diet diaries), as well as detailed physiological measures and sensitive cognitive tests of memory and learning, in women aged 25–45. We tested the hypothesis that greater saturated and trans fatty acid intakes, and a higher saturated fat to unsaturated fat ratio, would predict poorer performance of younger women on memory tasks expected to be sensitive to medial temporal lobe or hippocampal function.

## MATERIALS AND METHODS

### PARTICIPANTS

Women were recruited for this study from a larger sample (*n* = 68) who had been invited to take part in a study of polycystic ovary syndrome (PCOS), diet, and health, which included both women diagnosed with PCOS and matched non-PCOS controls (Barr et al., 2008). Recruitment for the larger study was via a self-help PCOS charity (Verity) in the UK and by local advertisement. Participants attended a university research center on one occasion and were invited to take part in this subsidiary study. It was explained that the only addition to the laboratory tasks involved was a 30-min computerized cognitive test session, which would occur while they waited to give a blood sample following an oral glucose tolerance test (OGTT). Forty-two women consented to take part, from whom 38 had complete data (*n* = 23 PCOS; *n* = 15 matched controls) and have been included in the results presented here. The diagnosis of PCOS was initially self-reported but then confirmed by their general practitioner, based on the presence of two of the following three features with the exclusion of other endocrine disorders: oligo-ovulation leading to oligomenorrhea, or anovulation leading to amenorrhoa; hyperandrogenism, clinical (hirsutism, male pattern alopecia, acne), and/or biochemical; polycystic ovaries (Barr et al., 2008). However, in this sample, there were no significant differences in either physiological (including endocrine), anthropometric (e.g., BMI, % body fat, waist–hip ratio, WHR) or cognitive function variables between women with and without diagnoses of PCOS, with the exception of slightly fewer errors on the National Adult Reading Test (NART; i.e., better verbal IQ or education) for the PCOS women. Thus, analyses were run on the 38 women as one group, but results were adjusted for NART errors (see below). Although this sample was small for an observational study, it compares favorably with sample sizes found in several of the experimental or intervention studies described above (Kretsch et al., 1997; Witte et al., 2009; Edwards et al., 2011; Holloway et al., 2011; Francis and Stevenson, 2013). This study was conducted according to the guidelines laid down in the Declaration of Helsinki and all procedures involving human participants were approved by Roehampton University and National Research Ethics Service (06/Q0104/130). Written informed consent was obtained from all participants.

### MEASUREMENT OF HEALTH BEHAVIORS

In the week preceding their attendance at the laboratory, habitual dietary intake and activity were assessed using a 7-day food and activity diary. Pedometers (Yamax Digi-walker SW-200) were provided to participants to wear for the same 7 days in order to characterize physical activity levels alongside the activity diary. Physical activity levels were calculated from the 7-day diary in order to assess the mean daily physical activity level of the participants. Activities were categorized into metabolic equivalents of energy (METs) as a multiple of basal metabolic rate. Categories were: light (<3 METs), moderate (3–6 METs) or high (>6 METs; Ainsworth et al., 2000). Food diaries were coded by a trained researcher and data were entered into a computerized dietary analysis package (Dietplan 6.3, Forestfield Software, Horsham, UK) to provide estimated daily energy and nutrient intakes.

### MEASUREMENT OF PHYSIOLOGICAL INDICATORS

Height, weight, and waist circumference were assessed via standardized protocols to ensure accurate measurement (Norgan, 2005). Total body adiposity was also measured under standardized conditions (Kushner, 1992) by tetra-polar bioelectrical impedance analysis using the Bodystat^®^ 1500 single-frequency body composition analyzer.

A fasting venous blood sample was taken for the assessment of fasting glucose and insulin levels, total cholesterol, high density lipoprotein (HDL) cholesterol, triacylglycerol (TAG), C-reactive protein (CRP), non-esterified fatty acids (NEFA), and plasminogen activator inhibitor-1 were measured using the Instrumentation Laboratory 650 (Milan, Italy) automated analyzer. Low-density lipoprotein (LDL) cholesterol levels were calculated using Friedewald’s equation (Friedewald et al., 1972). All hormones (free testosterone, androstenedione, insulin, cortisol, sex hormone binding globulin, dehydroepiandrosterone-sulfate, luteinizing hormone (LH), and follicle-stimulating hormone (FSH) were measured using an ELISA technique with consumables and reagents supplied by DGR Diagnostics (DRG Instruments GmbH, Germany). A 75-g glucose load for the OGTT was provided using Lucozade (GlaxoSmithKline, UK), following manufacturer’s recommended method, and plasma glucose and serum insulin measurements were taken at 0 and 120 min to determine fasting insulin sensitivity and 2-h glucose tolerance. The updated non-linear homeostatic model assessment (HOMA2) was used to calculate insulin resistance, β -cell function and insulin sensitivity using the Oxford Diabetes Trials Unit calculator (www.dtu.ox.ac.uk). The HOMA2 estimates correspond well to non-steady state estimates of β -cell function and insulin sensitivity derived from the hyperinsulinemic and hyperglycemic clamp (Levy et al., 1998). Insulin resistance was defined as a HOMA2 value >1.

### COGNITIVE TESTS

Three brief computerized tests were used from the CANTABeclipse (v3) suite of cognitive tests (Cambridge Cognition Ltd., Cambridge, UK), incorporating touch-screen responses. These tests were chosen because they are believed to be sensitive to dysfunction of the hippocampus and medial temporal lobe areas, which may be particularly susceptible to insult from dietary fats and effects of metabolic syndrome (Kanoski and Davidson, 2011). One is a verbal test, the other two are visuo-spatial in format.

#### Verbal recognition memory test (2 × 5 min)

In the verbal recognition memory (VRM) test, the participant was shown a list of 18 words on the screen, one at a time, which they were asked to remember as best they could. Then they were assessed as follows: (i) immediate recall: the number of words correctly recalled out loud immediately following the presentation (the participant faced away from the screen while their responses (correct words, new words, or repetitions) were scored on the computer by the investigator) (ii) immediate recognition: participants were presented with a randomized sequence of 36 words, half of which were on the original list to be memorized; as each word was presented, they were required to touch ‘yes’ or ‘no’ targets on the screen to indicate whether they recognized the word as being on the list to memorize (iii) delayed recognition: after a delay of 20 min, the word recognition procedure was repeated as before.

#### Delayed matching to sample (DMS) (10 min)

This test assessed both simultaneous and delayed recognition of novel patterns (delayed pattern matching, DMS); participants were shown a complex visual sample pattern and then (usually after a short delay) they were presented with an array of four patterns, and had to touch the pattern that was identical to the original sample pattern. Outcomes assessed response latencies, number of correctly chosen patterns, and the probability of an error after either correct or incorrect responding.

#### Paired associate learning task (PAL) (10 min)

This test assessed visuo-spatial memory and new learning, and can be used as a screening test for early signs of dementia or age-related memory decline. Boxes were displayed around the screen opening in randomized order, revealing either a novel pattern or no pattern. The sample patterns were then displayed in the middle of the screen, one at a time, and the participant had to touch the box where the pattern was originally located. If the participant made an error, the patterns were re-presented to remind the participant of their locations. The difficulty level increased through the test. In the clinical mode used here, the number of patterns revealed increased from 1, then 3, 6–8, with errors typically occurring once at least six pattern locations had to be recalled.

Outcomes for each level of difficulty covered the errors made by the participant, the number of trials required to locate the pattern(s) correctly, memory scores, and stages completed.

### PROCEDURE

Participants from the larger study of women with PCOS and matched controls attended the clinic at the University of Roehampton in the morning, having fasted overnight, for assessment on a range of physiological measures (described above). At this time they were asked to read the participant information on this supplementary study of cognitive function. Those volunteering to take part then signed their consent (*n* = 38).

As part of their assessments, all participants underwent an OGTT (see above), with blood draws 2 h apart. One hour after the glucose drink, they were seated in a quiet cubicle in front of a computer monitor with touch-screen response capability. Their distance from the screen was standardized as recommended by Cambridge Cognition Ltd. The sequence of tests was explained to them, and careful verbal instruction was given throughout, following the recommended text.

Prior to starting the CANTABeclipse test battery, the NART (NFER Nelson) was administered: this is believed to represent a form of stable verbal IQ that can act as an index of premorbid intelligence, although it also reflects educational attainment and socioeconomic status more generally (Crawford et al., 1988). Participants were presented with a series of cards one by one, with each card having a single word printed in capital letters. The participant was asked to pronounce each word out loud in the manner they believed was the correct pronunciation. The test was scored by summing the number of words that were not pronounced correctly (i.e., NART errors).

Next, the CANTAB eclipse tests were carried out, in the following order: VRM-immediate recall; VRM-immediate recognition; delayed matching to sample (DMS); visuo-spatial learning (PAL); VRM-delayed recognition. The battery lasted approximately 30 min, with a delay of 20 min between completion of the immediate and delayed VRM tests. After this, participants were thanked and debriefed, and returned to the clinic for completion of physiological assessments.

### DATA ANALYSIS

Variables were assessed for acceptably normal distribution by checking for skewness (< ±1) and extreme outliers by boxplots. The following variables were found to be skewed and were transformed by natural logarithm: fasting insulin, trans fatty acid intake, visuo-spatial learning total errors (eight shapes), VRM immediate free word recall.

Cognitive performance was significantly related to age (free word recall, DMS correct choice), activity (pedometer counts/day; visuo-spatial learning errors), and NART errors (word recall and recognition); therefore, these variables were controlled for using partial correlations or as covariates in subsequent multiple regressions.

Macronutrient intakes are, by definition, closely related to overall energy intake. Therefore, separate multiple linear regression mediation analyses with bootstrapping and covariates (Preacher and Hayes, 2008) were used to examine the possibility that macronutrient intake associations with cognition might be primarily mediated by overall energy intake. These were applied where overall energy intake was a significant predictor of cognitive performance after controlling for confounding variables by partial correlations. The outcomes of interest in these analyses were as follows: whether total energy intake had significant direct effects on cognition; whether the macronutrient had a significant direct effect on cognition after adjusting for the other variables in the model (all the above expressed as unstandardized regression coefficients (B) and their significance); and finally whether there was an indirect effect of the nutrient mediated via total energy intake (bootstrapping test; difference of mean effects from zero indicated by bias-adjusted 95% confidence intervals). The SPSS (v.19) syntax macro for these analyses was downloaded from the web (A. F. Hayes personal website, accessed 05.07.13: ).

## RESULTS

### PARTICIPANTS

All participants were female, aged between 25 and 45 years old. Four participants were current smokers, 12 were ex-smokers and 22 were non-smokers. Nine participants were non-native English speakers, but nevertheless fluent. Twenty-one participants reported taking dietary supplements regularly. Descriptive statistics for anthropometry are given in **Table 1**: 63% had BMIs (weight (kg)/height (m)^2^) within the healthy range, 24% were overweight (25 < BMI < 30), while 13% were obese (BMI = 30–35). Mean BMI was just under 25; by contrast, mean waist circumference was 82.6, thus being in the band (80–88 cm) associated with moderate risk for type-2 diabetes (T2D) and cardiovascular disease (The InterAct Consortium, 2012). The distribution of participants in these risk categories was 50% at low risk, 21% at moderate risk, and 29% at high risk. Not surprisingly, average waist:hip ratio and % body fat are both close to the border between low and raised risk for T2D.

**Table 1:** Participant characteristics (*n* = 38).

	Mean	SD	Min.	Max.
Age (years)	32.7	5.3	25	45
BMI (kg/m^2^)	24.9	4.5	18.4	35.0
Waist (cm)	82.6	12.4	64	109
Waist:hip ratio	0.79	0.07	0.68	0.99
Body fat (%)	31.4	7.1	20.0	46.2

Descriptive statistics for macronutrient intake are given in **Table 2**. These participants ate on average quite a low carbohydrate diet (44% of energy; **Table 2**), with relatively high protein (16.3%) and fat intakes (39.7%) compared with the recommendations for the UK population, i.e., 50% carbohydrate, 35% total fat, and 15% protein (Food Standards Agency, 2006). Similarly, the average saturated fat intake (13.9%) was higher than the recommended maximum of 11%. As with the anthropometric measures, these dietary data indicate that a significant proportion of the sample had unhealthy dietary practices and physiological indicators, which may be helpful for examining diet–cognition relationships.

**Table 2:** Average daily macronutrient and energy intakes from 7-day diet diaries (*n* = 38).

	Mean	SD	Min.	Max.
Total energy^a^ (MJ)	8.0	1.85	4.9	12.3
Protein (%)	16.3	2.8	12.1	23.1
Carbohydrate (%)	44.0	5.8	32.6	56.6
Fat (%)	39.7	6.5	27.1	58.6
Saturated fat (%)	13.9	3.7	7.8	25.6
Saturated fat (g)	28.2	9.4	14.3	48.6
Unsaturated fat (g)	42.0	10.7	21.6	63.8

a Including energy from alcohol.

*Macronutrient proportions are given as % total energy from food.*

### PHYSIOLOGICAL HEALTH

Both dietary fat intake and body fat % were associated with higher levels of peripheral indices related to metabolic syndrome, including total and LDL blood cholesterol, plasma C-reactive protein, blood pressure, and WHR, as indicated by significant positive correlations (**Table 3**). Waist circumference was very strongly associated with % body fat (*r* = 0.83, *p* < 0.001), but not significantly related to dietary fat intake (total fat *r* = 0.28, *p* = 0.08). Overall, the correlations support a stronger influence of saturated than unsaturated fats on these indices. However, it is notable that, whereas body fat was strongly associated with indicators of insulin resistance (fasting insulin and HOMA IR), no form of dietary fat intake was correlated to these measures. Furthermore, neither waist circumference, nor WHR was correlated with cognitive outcomes (controlling for age, activity and verbal IQ).

**Table 3:** Correlations (Pearson's *r*) between **%** body fat, dietary fat intakes and physiological indices related to metabolic syndrome (*n* = 38).

	% Body fat	Dietary fat (g)	Saturated fat (g)	Unsaturated fat (g)	Sat:UFA ratio	Trans fat (g)
Fasting insulin	0.59***	*–*	*–*	*–*	*–*	*–*
HOMA IR	0.49*	*–*	*–*	*–*	*–*	*–*
CRP	0.37*	0.28*	*–*	*–*	*–*	*–*
LDL-Chol.	0.39*	0.29^+^	0.38*	*–*	0.36*	0.32^+^
Total blood cholesterol	*–*	0.38*	0.39*	0.31^+^	*–*	*–*
BP (systolic)	0.39*	0.29*	0.29*	*–*	*–*	*–*
BP (diastolic)	0.46**	*–*	*–*	*–*	*–*	*–*
Waist:hip ratio	0.59***	0.38*	0.30^+^	0.31^+^	*–*	*–*

UFA, total unsaturated fatty acid intake; Sat:UFA ratio, ratio of total saturated to unsaturated fat intake; HOMA IR, homeostasis Model of assessment of insulin resistance; CRP, C-reactive protein; LDL-Chol., low-density lipoprotein blood cholesterol; BP, blood pressure.^+^*p* < 0.10 (two-tail), *p < 0.05, **p < 0.01, ***p < 0.001.

### COGNITIVE PERFORMANCE OUTCOMES

The performance outcomes were on average typical for relatively young and cognitively intact participants (**Table 4**; Cambridge Cognition Ltd.). Most outcomes showed a reasonable spread of performance across the sample for detecting associations with dietary factors, although the numbers of errors in the word recognition tests were quite restricted, and the mean % correct choices for the DMS task was high and slightly above normative data for this age group (93.4% vs. 87.5%), indicating the possibility of ceiling effects due to low task demands. The NART errors were on average quite high, suggesting moderate verbal IQ and educational status across the sample, although this may have been adversely affected by nine participants having English as a second language.

**Table 4:** Descriptive statistics for the main cognitive performance outcomes (*n* = 38).

Outcome	Mean	SD	Range
Visuo-spatial learning errors (PAL; eight shapes)	5.2	5.9	0–26
Words forgotten (VRM recall)	9.1	2.6	2–13
Word recognition errors (immediate)	1.8	1.8	0–6
Word recognition errors (delayed)	2.0	2.1	0–6
DMS correct choice (%; all delays)	93.4	5.6	73–100
DMS correct latency (s; all delays)	3.41	0.72	2.07–5.27
NART errors	20.0	6.6	5–36

### DIET AND COGNITIVE FUNCTION

#### Associations with dietary fats

Associations between dietary fats and measures of cognitive function were first examined by partial correlations controlling for age (pedometer) activity and verbal IQ (NART errors), as these background measures were associated with various cognitive outcomes. Higher intakes of total, saturated and trans fats, as well as greater saturated to unsaturated fat ratio, were associated with more errors on the visuo-spatial learning task (**Table 5**). Higher blood total cholesterol, itself associated with saturated fat intake (**Table 3**), was likewise associated with more visuo-spatial learning errors; however, by contrast, total unsaturated fat intake was not significantly related to this outcome (**Table 5**).

**Table 5:** Partial correlations^a^ between measures of memory function and dietary fat intakes or physiological indices in 25- to 45-year-old women (*n* = 38).

Test outcome	Total fat (g)	Saturated fat (g)	Unsaturated fat (g)	Sat:UFA ratio	Trans fat (g)	Blood cholesterol	Fasting insulin
Visuo-spatial learningerrors	0.38^*^	0.51^***^	––	0.48^**^	0.41^**^	0.32^*^	–
Words forgotten	0.40^*^	0.33^*^	0.46^*^	–	–	–	–
Word recog. errors – immediate	0.48^**^	0.38^**^	0.47^**^	–	–	–	0.32^*^
Word recog. errors – delayed	0.51^***^	0.47^**^	0.41^**^	–	–	–	0.36^*^

a Correlations were adjusted for verbal IQ (NART errors), age, and physical activity (pedometer counts). Nutrient intakes are from 7-day diet diaries. Only correlations significant at *p* < 0.05 or better are shown.

*Correlations for macronutrients in bold remained significant after testing for possible mediation by energy intake.*

* *p* < 0.05, **p < 0.01, ***p < 0.001.

Performance on the verbal memory task was also associated with fat intake, in that greater fat intake was predictive of more errors in both the immediate recall of words and for recognition of presented words immediately and after a 20 min delay (**Table 5**). However, in contrast to visuo-spatial learning errors, verbal memory errors were associated with greater intakes of both saturated and unsaturated fats, but not with the ratio nor with trans fats. Blood cholesterol did not predict verbal memory, but higher fasting insulin was associated with more word recognition errors.

Partial correlations were similarly conducted between macronutrient intakes, fasting insulin and outcomes for the DMS task: the only correlation found to approach significance was between the saturated to unsaturated fat ratio and mean correct latency (*r* = -0.30, *p* = 0.08), but as this was a negative correlation, implying shorter response times for more saturated fat intake vs. unsaturated fat, it may not be a reliable finding. In case the outcomes for DMS averaged over all trials reflected tasks that were insufficiently demanding, separate partial correlations were conducted for the trials with longer delays (4 and 12 s), but still no significant associations were seen (data not shown).

#### Associations with carbohydrate and protein intakes

Potential associations between intake of the other macronutrients, carbohydrate and protein, and cognitive performance, were also examined by partial correlations controlling for the same variables. There were no significant associations between carbohydrate intake and cognition (largest *r* = 0.29, *p* = 0.09, for delayed verbal recognition errors); however, protein intake was significantly positively correlated to word recognition errors (immediate, *r* = 0.41; delayed, *r* = 0.40, both *p* < 0.05) and to visuo-spatial learning errors (*r* = 0.34, *p* < 0.05), i.e., greater protein intake predicted worse verbal memory.

#### Mediation analyses examining the influence of total energy intake

The lack of associations with carbohydrate intake and cognition suggests that the other significant links are unlikely to be due to an effect of overall energy intake *per se*; nevertheless, this is an important concern, so to address the question whether these relationships between macronutrient intakes and cognition merely reflect a negative consequence of higher energy intake, which then mediates the apparent influence of macronutrients on cognition, multiple linear regression mediation analyses were conducted for each case where total energy was significantly correlated to performance (after controlling for age, activity, and verbal IQ), with the cognitive outcomes as dependent variables, and including age, activity, and verbal IQ scores as covariates.

The results for protein were as follows: for immediate verbal recognition errors, the mediation model did not show significant direct effects for either protein (*B* = 0.026) or total energy (as mediator; *B* = 0.001), and there was no indirect effect of protein mediated by energy intake (95% CI overlapping with 0: bootstrapping bias-corrected 95% CI = -0.027 to 0.073), despite a significant overall adjusted effect of protein on recognition errors (**Table 6**). For delayed recognition errors, total energy intake did have a significant direct effect on performance (*B* = 0.002, *p* < 0.05); however, protein did not have a significant direct effect, and although the overall model was significant (**Table 6**), the indirect effect of protein via total energy intake as mediator was not significant (bootstrapping bias-corrected 95% CI = -0.005 to 0.092). For visuo-spatial learning errors (eight shapes), the mediation model did not show significant direct effects for either protein or total energy (as mediator), and there was no indirect effect of protein mediated by energy intake (bootstrapping 95% CI = -0.010 to 0.030), despite a significant overall adjusted effect of protein on recognition errors (**Table 6**: *B* = 0.018, *p* = 0.05). In summary, it would appear that protein does not reliably predict verbal or visual memory, when covariates and energy intake are adjusted for; however, this also cannot be attributed simply to a mediating effect of energy intake.

**Table 6:** Adjusted *R*^2^ values and significance for the overall multiple regression models examining potential mediation by total energy intake of the macronutrient-cognition association, including age, verbal IQ and activity as covariates.

Test outcome	Total fat (g)	Saturated fat (g)	Unsaturated fat (g)	Trans fat (g)	Protein (g)
Visuo-spatial learning errors	0.20^*^	0.31^**^	^-^	0.25^*^	0.18^*^
Words forgotten	0.34^**^	0.29^**^	0.35^**^	–	–
Word recog. errors – immediate	0.34^**^	0.31^**^	0.34^**^	–	0.31^**^
Word recog. errors – delayed	0.33^**^	0.34^**^	0.32^**^	–	0.31^**^

*Empty cells indicate that there was no partial correlation between the nutrient and the cognitive outcome, hence no mediation was tested.*

* *p* < 0.05, **p < 0.01.

The mediation results for total fat intake were as follows: for immediate verbal free recall, energy intake did not have a significant direct effect on number of words forgotten (*B* = -0.0001), whereas total fat intake was directly associated with words forgotten (*B* = 0.006, *p* < 0.05)). There was no evidence for a significant indirect effect of fat intake on words forgotten mediated by energy intake (bootstrapping 95% CI = -0.006 to 0.002). By contrast, for both immediate and delayed recognition errors, there were no significant direct effects of either fat (imm. *B* = 0.029; delayed *B* = 0.027) or energy intake (imm. *B* = 0.0005; delayed *B* = 0.001), and no indirect effects of fat mediated by energy intake (bootstrapping 95% CI: imm. = -0.032 to 0.046; delayed = -0.026 to 0.068). For visuo-spatial learning errors, neither total fat (*B* = 0.015) nor energy intake (*B* = 0.0001) had a significant direct effect. There was also no evidence for a significant indirect effect of fat intake on visuo-spatial learning errors mediated by energy intake (bootstrapping 95% CI = -0.007 to 0.002). In summary, for immediate word recall, but not for word recognition or visuo-spatial learning errors, higher total fat intake was significantly associated with more words forgotten independently of energy intake.

The mediation results for saturated fat intake were as follows: for immediate verbal free recall, neither energy intake nor saturated fat intake had a significant direct effect on number of words forgotten (energy *B* < 0.0000; sat. fat *B* = 0.008). There was no evidence for a significant indirect effect of saturated fat intake on words forgotten mediated by energy intake (bootstrapping 95% CI = -0.005 to 0.007). Likewise, for both immediate and delayed recognition errors, there were no significant direct effects of either saturated fat (imm. *B* = 0.033; delayed *B* = 0.050) or energy intake (imm. *B* = 0.0012; delayed B = 0.0016), and no indirect effects of saturated fat mediated by energy intake (bootstrapping 95% CI: imm. = -0.009 to 0.117; delayed = -0.020 to 0.123). By contrast, for visuo-spatial learning errors, saturated fat had a significant direct effect (*B* = 0.045, *p* < 0.05), whereas energy intake did not (*B* < 0.0000). There was no evidence for a significant indirect effect of saturated fat intake on visuo-spatial learning errors mediated by energy intake (bootstrapping 95% CI = -0.020 to 0.023). In summary, although saturated fat was overall associated with poorer performance on word recall and recognition (**Table 6**), these effects were no longer significant when energy intake was adjusted for; however, this also could not be attributed simply to a mediating effect of energy intake. By contrast, higher saturated fat intake was significantly associated with more visuo-spatial learning errors, independently of any mediating influence of energy intake.

The mediation results for unsaturated fat intake were as follows: for immediate verbal free recall, energy intake did not have a significant direct effect on number of words forgotten (*B* = -0.0001), whereas unsaturated fat intake was directly associated with words forgotten (*B* = 0.011, *p* < 0.05). There was no evidence for a significant indirect effect of unsaturated fat intake on words forgotten mediated by energy intake (bootstrapping 95% CI = -0.007 to 0.004). However, for immediate recognition errors, there were no significant direct effects of either unsaturated fat (*B* = 0.049) or energy intake (*B* = 0.0009), and no indirect effects of fat mediated by energy intake (bootstrapping 95% CI = -0.021 to 0.077). By contrast, for delayed recognition, the direct effect of energy intake was significant (*B* = 0.0018, *p* < 0.05), whereas the direct effect of unsaturated fat was not (*B* = 0.029). Moreover, there was evidence for an indirect effect of unsaturated fat on delayed word recognition significantly mediated by energy intake (bootstrapping 95% CI = 0.002–0.129).

In summary, when possible mediation by energy intake was tested for, there were opposing associations between unsaturated fat and verbal memory: for immediate word recall, higher unsaturated fat intake was associated with more words forgotten independently of energy intake. By contrast, unsaturated fat intake was not associated with immediate word recognition, and for delayed word recognition, the association was clearly mediated by energy intake. As regards the association between the saturated to unsaturated fat ratio and visuo-spatial learning errors, it is not meaningful to test this for mediation by total energy intake, as the ratio is independent of how much energy is eaten.

#### Associations with mono- and polyunsaturated fat intake

Given the unexpected evidence for a detrimental effect on verbal memory (free word recall) of higher intakes of unsaturated fats, we examined whether this association was specific to MUFA vs. PUFA fat intake, by applying the mediation analyses to intake of these fat types separately. For PUFA intake, there was no significant direct effect for either energy intake (*B* < 0.0000) or PUFA intake (*B* = 0.017, *p* > 0.10) on words forgotten (overall model adjusted *R*^2^ = 0.29, *p* < 0.01). However, for MUFA intake, whereas there was no significant direct effect of energy intake, the direct effect of MUFA intake on words forgotten remained significant (*B* = 0.017, *p* < 0.05; partial correlation excluding energy intake = 0.45). For both PUFA and MUFA, there was no evidence of indirect effects mediated by energy intake (95% CI: PUFA = -0.007 to 0.015; MUFA = -0.012 to 0.005). The partial correlation between MUFA intake and words forgotten remained significant and was little altered after additionally controlling for whether or not the participant was taking dietary supplements (0.42, *p* < 0.05).

The mediation results for the positive association between trans fatty acid intake and visuo-spatial learning errors were as follows: the direct effect of trans fats on visuo-spatial learning errors was marginally significant (*B* = 0.900, *p* = 0.05), whereas there was no direct effect of energy intake (*B* = 0.0004), and there was no evidence for mediation of the trans fat effect by energy intake (bootstrapping 95% CI = -0.019 to 0.787). The overall regression model was significant (**Table 6**).

## DISCUSSION

This study used detailed dietary measures together with sensitive tests of cognition to test the hypothesis that higher habitual dietary fat intake may be associated with poorer memory function in women in early to middle adulthood. The findings largely supported this hypothesis: higher fat intake was associated with worse performance on tests of visuo-spatial learning and verbal (VRM) learning and memory, when adjusting for the influence of age, activity, and verbal IQ. Furthermore, for saturated fat and trans fatty acid intakes, and for the ratio of saturated to UFA intake, these associations were shown to be independent of overall energy intake. By contrast, partial correlations between higher protein intake and worse memory performance were no longer significant when adjusted for energy intake, although mediation of these effects of protein by energy intake *per se* was not shown to be significant in this relatively small sample. Carbohydrate intake was not significantly associated with any of the cognitive performance tests.

With respect to brain regions that may be most affected, this study was limited to tests thought to be sensitive to medial temporal lobe and particularly hippocampal function, i.e., tasks involving visual and verbal memory and learning associations. Whereas two of the three tests administered (visuo-spatial learning and verbal recall/recognition) revealed significant associations between dietary fats and performance, the DMS test did not. The DMS test shares similarities with the visuo-spatial learning task (PAL), in that both are tests of short-term visual memory using pattern stimuli, and both would be expected to be sensitive to function of the medial temporal lobe areas of the cerebral hemispheres. However, the visuo-spatial learning task more explicitly involves new learning, and at the level of eight shapes for which recall location is needed the test is more challenging than DMS, which involves detecting a target stimulus among only four choices. Indeed, with correct choices made on an average of 93% of trials (somewhat higher than the normative score for this age group; Cambridge Cognition, Ltd.), DMS may not have been sensitive enough to subtle impairments of medial temporal lobe function in this relatively healthy and young sample. Nevertheless, our conclusions are limited by the lack of tests designed specifically to assess frontal lobe function, for example. In addition, test sensitivity may have been affected by administering the 75-g glucose drink prior to testing, because it is well-established that glucose loads can improve performance, particularly on tasks involving medial temporal lobe function, albeit usually in lower doses (Smith et al., 2011). Thus, whereas this manipulation served to ensure that all participants were in the same acute nutritional state, it may have somewhat reduced the variation in performance across participants. This would imply that the actual impact of habitual fat intake on cognition could be greater than that seen in this paradigm.

These findings are in line with epidemiological data in the elderly (Kalmijn et al., 2004; Morris et al., 2004; Gillette Guyonnet et al., 2007; Eskelinen et al., 2008; Okereke et al., 2012), but additionally suggest that dietary factors, e.g., high saturated fat, may lead to cognitive impairment in much younger women. This interpretation is limited by the cross-sectional nature of the data, and the small sample size, but unlike many larger population studies, we used detailed 7-day dietary diaries, and cognitive tests designed to assess hippocampal function that may be particularly sensitive to nutritional insult. Moreover, this is supported by notable effect sizes, with the overall regression models explaining 20–35% of the variance. Also, we only examined linear relationships, and it is possible the non-linear modeling would produce even greater effect sizes, i.e., there could be threshold effects. Another strength of the study was that all women were tested in the same acute nutritional state, i.e., following a glucose load after overnight fasting.

However, one finding was unexpected: higher UFA intake was associated specifically with poorer verbal recall memory, independently of energy intake. This contrasts with verbal recognition performance, for which the association with UFA intake was found to be indirect and mediated by energy intake. Separate examination of MUFA and PUFA associations with memory suggested that MUFA intake, but not PUFA intake, was linked to poorer verbal recall, and this could not be accounted for by overall energy intake. It is important to examine a possible independent effect of overall energy intake, as a recent intervention in the elderly found that energy restriction (or at least weight loss) improved verbal memory, possibly via increased insulin sensitivity or reduced inflammation (Witte et al., 2009). Of interest also in that study, raising intake of MUFA did not benefit memory, although PUFAs were not increased, and numbers were limited to 20/group. Such beneficial cognitive effects of weight loss may be specific to verbal memory, suggesting a role for hippocampal changes (Kretsch et al., 1997).

Of course, there are several observational studies, both cross-sectional and longitudinal, that support positive rather than negative associations between MUFA and cognitive function (Solfrizzi et al., 2006; Naqvi et al., 2011; Okereke et al., 2012). Moreover, enhanced MUFA intake was found to be associated with better cognition in a randomized controlled trial (Martinez-Lapiscina et al., 2013). MUFA intake in a non-Mediterranean sample such as this may differ in the influence of unadjusted confounders than that in a Mediterranean culture; nevertheless, other studies have found significant cognitive benefits from higher PUFA but not MUFA intake, in both Mediterranean (Psaltopoulou et al., 2008) and non-Mediterranean cultures (Samieri et al., 2013). Furthermore, whereas PUFA intake in a London population was protective against cardiovascular disease, MUFA intake was not (Clarke et al., 2009). Even more critically, a recent 4-year prospective epidemiological study of aging in Australians found that high MUFA (and energy) intake predicted mild cognitive impairment (Cherbuin and Anstey, 2012). These findings and those reported here suggest that associations between MUFA intake and memory function need careful examination in a variety of populations. It is possible that in certain cultures there is residual confounding between MUFA intake and another ingredient, or indeed behavior, which could adversely affect memory function. Even so, interpretation of such findings, including our own, is complicated by the lack of distinction between different forms of fatty acid in these studies: in particular, among PUFA, a higher proportion of n-6 vs. n-3 essential fatty acids may be an important predictor of adverse outcomes for brain as well as cardiovascular health (Loef and Walach, 2013), perhaps because of respective pro-inflammatory vs. anti-inflammatory actions of their eicosanoid products (Calon and Cole, 2007). This may be particularly important given the typically very high ratio of n-6 to n-3 fatty acids (e.g., at least 15:1) in modern Western diets (Loef and Walach, 2013), and increasingly so in some developing countries particularly in Asia (Misra et al., 2010).

Beyond the dietary fat associations with memory, there was limited evidence for relationships between physiological indices and cognition. Even in these younger women, body fat levels were associated with physiological indices of risk for T2D and cardiovascular disease, such as LDL cholesterol, CRP, blood pressure and insulin resistance (HOMA IR and fasting insulin), as well as WHR. Nevertheless, these physiological measures were, by and large, unrelated to cognitive function in this sample – the exception being higher fasting insulin and total cholesterol, which had moderate associations with poorer word recognition and visuo-spatial learning errors, respectively. This might reflect the relatively healthy status of this sample, as dietary fat type is known to affect indices of metabolic syndrome (Clarke et al., 2009), and other research has shown cognitive impairments associated with T2D and abdominal fat (Isaac et al., 2011), even in adolescents (Schwartz et al., 2013), although this relationship may be less clear in women (Kanaya et al., 2009).

The mechanisms by which dietary fats might impair the activity of neurons in the hippocampus are not known for certain; however, several pathways have been proposed. For example, suppression of neurogenesis may be important, i.e., preventing the formation of new neurons – which occurs only in the hippocampus and one other brain region (subventricular zone) – as high-fat diets reduce brain derived neurotrophic factor (BDNF) in rats, which is believed to be a key factor in neurogenesis (Molteni et al., 2002). There is experimental evidence in laboratory models that fatty acids could modulate adult hippocampal neurogenesis and thus affect learning and memory (Stangl and Thuret, 2009; Zainuddin and Thuret, 2012). Similarly, high levels of saturated and trans fatty acids, and low PUFA intake, have been associated with increased cholesterol and inflammatory markers that themselves are linked to impairment of neurogenesis and cognition as well as cardiovascular disease (Clarke et al., 2009; Marioni et al., 2009). By contrast, PUFAs, especially n-3 fatty acids, essential components of neuronal cell membranes as well as precursors for anti-inflammatory eicosanoids, appear to have protective effects (Vercambre et al., 2009), promoting neurogenesis in the hippocampus (Cao et al., 2009). Other mechanisms may include changes in insulin sensitivity of hippocampal neurons, and local permeability of the blood–brain barrier (McNeilly et al., 2011; Davidson et al., 2012; Pancani et al., 2013).

In summary, we found evidence that high intake of saturated and trans fats in particular, and possibly MUFA, may adversely affect memory and learning even in relatively young women. Our results are confined to women, and our sample had a high proportion diagnosed with PCOS: thus, extrapolating to the general population is not yet justified. Results are also limited to the tasks employed, and will benefit from replication and extension in a larger sample in which relevant genotyping could also be deployed (e.g., ApoA4, FADS), and including a wider range of tests, to determine their reliability and specificity. Moreover, this was an observational cross-sectional study, and we cannot be certain about causal interpretations. Nevertheless, as they stand, they have important implications for public health messages: arguably, younger adults may find messages linking diet to learning and memory to be more compelling than those aimed at promoting cardiovascular health, even if the health behavior requirements are the same. Furthermore, in elderly patients already suffering from dementia, dietary interventions have generally not been successful (Fotuhi et al., 2009). Thus, intervention studies conducted in younger participants before overt cognitive decline has become apparent, and which carefully disconfound variables such as socioeconomic status, education, and other health behaviors (Gillette Guyonnet et al., 2007) are more likely to produce effective recommendations to alter the unaffordable trajectory of diet-mediated cognitive decline.

## Conflict of Interest Statement

The authors declare that the research was conducted in the absence of any commercial or financial relationships that could be construed as a potential conflict of interest.

## AUTHOR CONTRIBUTIONS

E. Leigh Gibson was primary author of this manuscript, and designed and executed the cognitive testing component, and analyzed those data. Suzanne Barr was responsible for recruitment and assessment of participants for dietary and physiological measures, as well as data analysis of dietary diaries. Yvonne M. Jeanes designed the larger observational study, initiated recruitment, and carried out data collection and analysis.
